# Bladder Cancer Segmentation in CT for Treatment Response Assessment: Application of Deep-Learning Convolution Neural Network—A Pilot Study

**DOI:** 10.18383/j.tom.2016.00184

**Published:** 2016-12

**Authors:** Kenny H. Cha, Lubomir M. Hadjiiski, Ravi K. Samala, Heang-Ping Chan, Richard H. Cohan, Elaine M. Caoili, Chintana Paramagul, Ajjai Alva, Alon Z. Weizer

**Affiliations:** 1 Departments of Radiology;; 2 Internal Medicine, Hematology-Oncology; and; 3 Urology, Comprehensive Cancer Center, University of Michigan, Ann Arbor, Michigan

**Keywords:** computer-aided diagnosis, deep-learning, CT, bladder cancer, treatment response, segmentation, level set

## Abstract

Assessing the response of bladder cancer to neoadjuvant chemotherapy is crucial for reducing morbidity and increasing quality of life of patients. Changes in tumor volume during treatment is generally used to predict treatment outcome. We are developing a method for bladder cancer segmentation in CT using a pilot data set of 62 cases. 65 000 regions of interests were extracted from pre-treatment CT images to train a deep-learning convolution neural network (DL-CNN) for tumor boundary detection using leave-one-case-out cross-validation. The results were compared to our previous AI-CALS method. For all lesions in the data set, the longest diameter and its perpendicular were measured by two radiologists, and 3D manual segmentation was obtained from one radiologist. The World Health Organization (WHO) criteria and the Response Evaluation Criteria In Solid Tumors (RECIST) were calculated, and the prediction accuracy of complete response to chemotherapy was estimated by the area under the receiver operating characteristic curve (AUC). The AUCs were 0.73 ± 0.06, 0.70 ± 0.07, and 0.70 ± 0.06, respectively, for the volume change calculated using DL-CNN segmentation, the AI-CALS and the manual contours. The differences did not achieve statistical significance. The AUCs using the WHO criteria were 0.63 ± 0.07 and 0.61 ± 0.06, while the AUCs using RECIST were 0.65 ± 007 and 0.63 ± 0.06 for the two radiologists, respectively. Our results indicate that DL-CNN can produce accurate bladder cancer segmentation for calculation of tumor size change in response to treatment. The volume change performed better than the estimations from the WHO criteria and RECIST for the prediction of complete response.

## Introduction

Bladder cancer accounts for 5% of all new cancers in the USA, and it is the fourth most common cancer in men. The American Cancer Society estimates that in 2016, 76 960 (men, 58 950; women, 18 010) new cases of bladder cancer will be diagnosed in the USA, with 16 390 (men, 11 820; women, 4570) deaths (1). Early treatment of bladder cancer is important to reduce morbidity and mortality, as well as reduce costs.

The standard treatment method for bladder cancer involves radical cystectomy of the bladder; however, ∼50% of the patients who have under gone cystectomy and were considered to have only locally invasive cancer at the time of the surgery develop metastatic disease within 2 years and subsequently die because of the disease (2). This may be because of the presence of micrometastatic disease or the presence of neoplasms that have spread to perivascular tissue that went undetected at the time of treatment. Neoadjuvant chemotherapy improves resectability of large tumors, and it is beneficial for the treatment of micrometastases before radical cystectomy (3–5). The treatment regimen with methotrexate, vinblastine, doxorubicin, and cisplatin decreases the chance of finding residual cancer after cystectomy compared with treatment with cystectomy alone, and increases the survival of patients with locally advanced bladder cancers (6, 7). However, the side effects of this treatment are severe, which include neutropenic fever, sepsis, mucositis, nausea, vomiting, malaise, and alopecia (8). Because there are no reliable methods at present to predict a patient's response to chemotherapy, it is possible that patients experience these side effects while enduring treatment that may or may not achieve the desirable benefits. Therefore, early assessment of the bladder cancer treatment response is important, allowing clinicians to put a timely stop to unbeneficial treatment. This will help reduce patient morbidity and increase their quality of life. It may also preserve a patient's physical conditions and allow them to pursue alternative treatment that may be more beneficial.

The response to treatment can be measured via pathological information from the resected bladder after cystectomy or other surgical procedures such as transurethral resection of bladder tumor. However, because these patients are receiving chemotherapy, surgery may not be an ideal method for assessing treatment response. The patients are weak because of chemotherapy, and therefore, surgery is not recommended during their chemotherapy treatment; noninvasive evaluation is preferable. Image-based evaluation, using either computed tomography (CT) or magnetic resonance imaging images, can noninvasively visualize the tumor during the chemotherapy treatment. Specific features from these images, known as radiomic features, may be extracted and analyzed to determine tumor properties.

The clinical estimation of the tumor size and its response to treatment is based on the World Health Organization (WHO) criteria (9) and the Response Evaluation Criteria in Solid Tumors (RECIST) (10). As per the WHO criteria, the longest diameter of a tumor and its perpendicular diameter are measured. The response to treatment is defined as the percentage reduction of the products of the 2 diameters between the pre- and post-treatment measurements. The RECIST criteria use the percentage reduction of the longest diameter between the pre- and post-treatment measurements. Both methods can be inaccurate and can have large inter- and intraobserver variations, particularly for tumors with irregular and complex shapes (11). As RECIST criteria and WHO criteria involve only 1-dimensional (1D) and 2-dimensional (2D) measurements, respectively, the volumetric (3-dimensional [3D]) information from a CT scan is not fully used, and it is possible that the 3D information may provide better response evaluation.

The gross tumor volume (GTV) can be effectively measured in CT images, and it can predict outcomes of bladder cancers (12). For an accurate GTV measure from the CT images, the bladder tumor in the images needs to be delineated section by section; however, this is a time- and labor-intensive procedure, and thus, the burden of the additional workload on the radiologists cannot be ignored. Computerized segmentation tools that can automatically or semiautomatically delineate the tumors from its surroundings would greatly reduce the additional workload. In our previous preliminary study, we have shown that our method for computerized segmentation of bladder tumors, auto-initialized cascaded level sets (AI-CALS), can reliably produce 3D segmentation for various bladder tumors (13), and that the volume estimates more accurately predict the complete response to treatment compared with the WHO and the RECIST criteria on small data sets (14).

Although the 3D volumetric measurement by AI-CALS provides better estimates than the 1D and 2D estimates in tumor size changes, the segmentation method still needs improvement in many cases given the various tumor shapes and characteristics in the patient population. Here, we explored the application of deep-learning convolution neural network (DL-CNN) to the segmentation of bladder tumors. Convolution neural networks (CNNs) have been used previously to classify patterns in medical images for use with computer-aided detection, particularly for microcalcification and mass detection in mammograms (15–23). The training set used in these applications was typically small, in general, <500 samples. With advances in computation power, it has become practical to design CNNs with very large and complex architectures that require a massive number of training samples to solve more challenging pattern recognition problems. The DL-CNN using graphics processing units has been successful in classifying natural scene images using a large training set. Krizhevsky et al. (24, 25) showed that high classification accuracy can be achieved using DL-CNN on the ImageNet ILSVRC-2010 and ILSVRC-2012 data sets (26) and the CIFAR-10 data set (27). DL-CNN has also been successfully used for computer-aided detection in medical imaging (28). We have previously applied DL-CNN to the segmentation of whole bladders in CT images (29); however, the segmentation of the tumors is more difficult because contrast material is generally not used in CT for patients undergoing chemotherapy, resulting in low contrast between the tumor and the inside of the bladder.

In this pilot study, we applied DL-CNN to bladder lesion segmentation. For this task, the DL-CNN was trained to recognize the patterns in the regions that were inside and outside of the bladder lesion and generate a lesion likelihood map. Minor refinement on the likelihood map was performed by level sets to obtain the segmented boundaries of the bladder cancer.

The bladder cancer segmentation performance of the DL-CNN and the AI-CALS was quantitatively compared with the radiologists' manual outlines. The cancer volumes were calculated from the segmented tumor boundaries, and the GTV change in response to neoadjuvant chemotherapy was calculated. The results obtained from the DL-CNN were compared with the results obtained using our previous AI-CALS segmentation method, radiologist's manual outlines, and response estimation using the WHO and the RECIST criteria.

## Methods

### Data Set

A data set of 62 cases was collected retrospectively from the Abdominal Imaging Division of the Department of Radiology at the University of Michigan with Institutional Review Board approval for this pilot study. All patients in the data set underwent CT examination before and after chemotherapy, and subsequently, underwent cystoscopy, biopsy, or radical cystectomy. The CT scans used in this study were acquired in our clinic with GE Healthcare LightSpeed MDCT scanners (GE Healthcare, Waukesha, Wisconsin). The images were acquired using 120 kVp and 120–280 mA and reconstructed at a section interval of 0.625, 1.25, 2.5, or 5 mm, with pixel sizes ranging from 0.586 to 0.977. The data set contained 64 tumors forming 74 temporal pairs, and 27% (17/62) of the patients showed stage pT0 after surgery, indicating complete response to treatment.

A reference standard for the computerized segmentation was obtained via 3D hand-segmented contours of the bladder tumors in the pre- and post-treatment CT of the 62 cases. A radiologist with 27 years of experience in CT bladder cases identified and marked focal tumor locations within the CT scans. The radiologist also manually outlined the full 3D contour for all cases (reference standard 1), and measured the longest diameter and its perpendicular diameter using a graphical user interface we have developed. The second radiologist with 17 years of experience in CT bladder cases also manually outlined the bladder tumors in the pre- and post-treatment CT for a subset of 29 cases (reference standard 2), and measured the longest diameter and its perpendicular diameter independently following the WHO criteria and RECIST criteria for all 62 cases.

### DL-CNN Training

**Figure 1.**
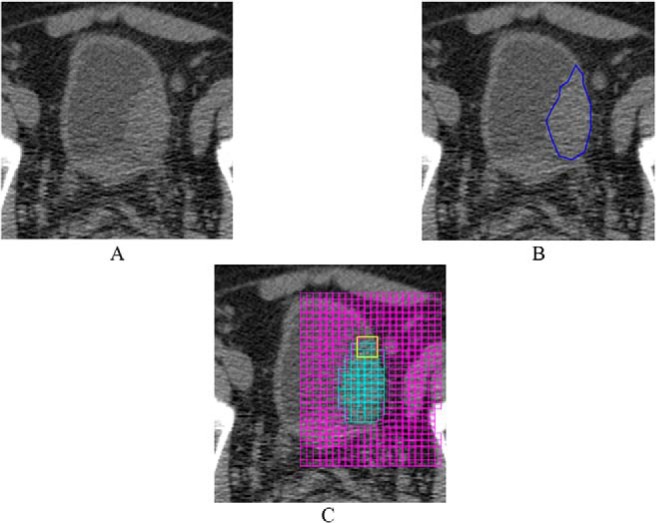
An axial section of a pre-treatment computed tomography (CT) scan from a training case. Cropped CT section centered at the bladder (A). Radiologist's hand-outline of the cancer overlaid on the CT section (B). Regions of interest (ROIs) extracted from this section (C). The yellow ROI shows the size of a 16- × 16-pixel ROI. The ROIs are partially overlapping. The blue ROIs are labeled as being inside the bladder cancer. The pink ROIs are labeled as being outside the bladder cancer for training the deep-learning convolution neural network (DL-CNN).

**Figure 2.**
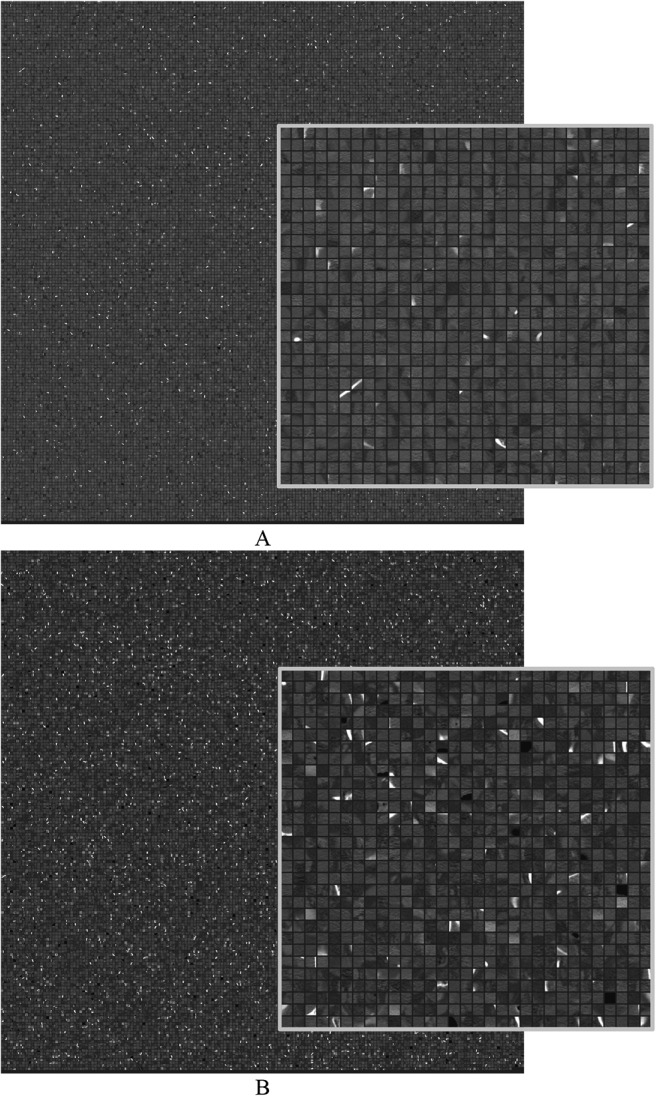
Composite images of the 47 000 ROIs from the training set used to train the DL-CNN. Each ROI is 16 × 16 pixels. ROIs labeled as being inside bladder cancers (A). ROIs labeled as being outside bladder cancers (B). A portion of each composite image is enlarged to show the typical ROIs in each class.

Our research work on whole bladder segmentation using DL-CNN was expanded further to bladder tumor segmentation. The DL-CNN by Krizhevsky et al. called cuda-convnet (24, 25) was used. The neural network was trained to classify regions of interests (ROIs) on 2D sections as being either inside or outside of the bladder cancer. Details on the DL-CNN can be found in the literature (29). The DL-CNN was trained with the pretreatment scans of the cases. For each axial section of the cases, a large number of overlapping 16- × 16-pixel ROIs were extracted from the region including the cancer marked by the radiologist. If >80% of an ROI was within the hand-outlined bladder cancer, the ROI was labeled as being inside of the cancer, whereas the ROI had to be completely outside of the cancer for it to be classified as being outside of the cancer. ROIs not labeled as either inside or outside of the cancer were excluded. Figure 1 shows an example of ROIs obtained from a CT section. The number of ROIs within the 2 classes was balanced, resulting in ∼65 000 ROIs. Figure 2, A and B shows examples of ROIs inside and outside of the bladder cancer, respectively, that were used to train the DL-CNN.

The network architecture used in this study consists of the following 5 main layers: 2 convolution layers, 2 locally connected layers, and 1 fully connected layer. The first convolution layer consists of 64 kernels with a size of 5 × 5 pixels. The output of this layer is pooled, normalized, and input into the second convolution layer that also consists of 64 kernels with a size of 5 × 5 pixels. The output of this layer is also pooled, normalized, and input to the first locally connected layer that has 64 kernels with a size of 3 × 3 pixels. The second locally connected layer has 32 kernels with a size of 3 × 3 pixels and output to the fully connected layer. The fully connected layer outputs 2 values to a softmax layer that converts the values to a range from 0 to 1. The output of the DL-CNN can be interpreted as the likelihood of an input ROI being classified into 1 of the 2 categories. The neural network was trained for 1500 iterations, which was sufficient for the classification error rate to converge to a minimum and remained stable. Leave-one-case-out cross-validation was used for this study. In each of the leave-one-case-out partitions, all ROIs associated with a case were removed, and the DL-CNN was trained using the remaining ROIs. The training of the DL-CNN for 1 partition took ∼1.5 hours on average using an Nvidia Tesla K20 graphics processing unit.

### Bladder Cancer Likelihood Map Generation Using DL-CNN

**Figure 3.**
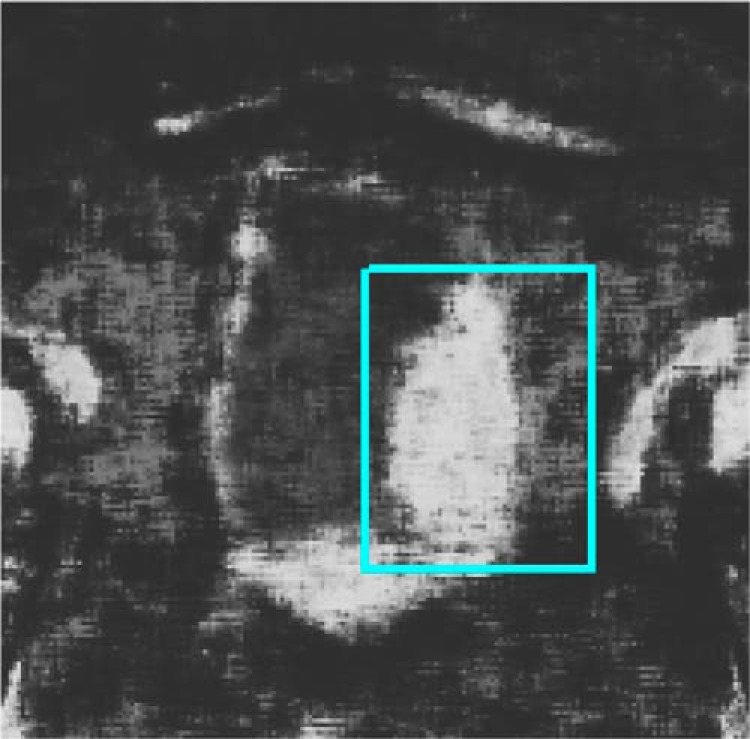
Bladder cancer likelihood map of the CT section shown in Figure 1. Regions that are highly likely to be bladder cancer have higher intensity values. The volume of interest (VOI) that was used for this lesion is shown in blue. For demonstration purposes, the bladder cancer likelihood map was generated in the region around the entire bladder.

For each leave-one-case-out partition, the trained DL-CNN network was applied to the left out case to generate the bladder cancer segmentation likelihood map. A bladder cancer likelihood map was generated by applying the trained DL-CNN to a volume of interest (VOI) of a CT scan that contained the bladder tumor to be segmented. In this study, a VOI that approximately enclosed the bladder cancer was manually marked in each CT scan. For every voxel within the VOI, an ROI of 16 × 16 pixels in size centered at the voxel was automatically extracted from the corresponding axial section and input into the DL-CNN, which estimated a likelihood score that the voxel was inside the tumor. After the likelihood values were estimated for all voxels in the VOI, the likelihood values on each section constituted a 2D likelihood map on the axial section, and the stack of 2D likelihood maps provided the 3D map for the VOI. Figure 3 shows the bladder cancer likelihood map for the CT section shown in Figure 1. The DL-CNN was applied to the CT scan for both the pre- and post-treatment scans for each bladder cancer case.

### Bladder Cancer Segmentation from Likelihood Map

As seen in the example of Figure 3, the likelihood map identifies the bladder tumor region very well, but the tumor boundary is not sharply demarcated. The level sets, therefore, are used to perform minor refinements to the contour. First, a binary cancer mask, *DL*_*Mask*_, is generated by applying the following equation to every pixel of every section of the likelihood map:

(1) $$DL_{Mask}\left(x,y\right)=\left\{ \begin{array}{l} 1,\;\;\;DL_{Score}\left(x,y\right)\geq \rho \\ 0,\;\;\;DL_{Score}\left(x,y\right)<\rho \end{array}\right.$$

where *DL*_*Mask*_(*x, y*) is the pixel value on the cancer mask at the coordinates (*x, y*) of a section, *DL*_*Score*_(*x, y*) is the bladder cancer likelihood score at the coordinates (x, y), and ρ is the likelihood score threshold. The value of ρ was experimentally determined to be 0.60 to generate reasonable binary masks in comparison to the radiologist's manual segmentation. A morphological dilation filter with a spherical structuring element of 2 voxels in radius, a 3D flood fill algorithm, and a morphological erosion filter with a spherical structuring element of 2 voxels in radius were used to smooth the cancer mask and connect neighboring components to extract an initial segmentation surface, ϕ_0_(*x*).

The initial segmentation surface was refined using level sets. For this study, the level set uses the following equation:

(2) $$\left\{ \begin{array}{l} \frac{\partial }{\partial t}\Psi \left(x\right)=-\alpha A\left(x\right)\nabla \Psi \left(x\right)-\beta P\left(x\right)\left|\nabla \Psi \left(x\right)\right|\\ \;\;\;\;\;\;\;\;\;\;\;\;\;\;\;\;\;\;\;\;+\gamma \kappa \left(x\right)\left|\nabla \Psi \left(x\right)\right|\\ \Psi \left(x,n=0\right)=\phi_{0}\left(x\right) \end{array}\right.$$

where α, β and γ are the coefficients for the advection, propagation, and curvature terms, respectively; *A*(*x*) is a vector field image that drives the contour toward regions of high gradient; *P*(*x*) is a scalar speed term between 0 and 1, causing the contour to expand at the local rate; and $\kappa \left(x\right)=div\left(\frac{\nabla \Psi \left(x\right)}{\left|\nabla \Psi \left(x\right)\right|}\right)$ is the mean curvature of the level set at point x. The symbol ∇ denotes the gradient operator and *div* is the divergence operator (30); *n* is the number of iterations.

A 3D level set with a predefined set of parameters is applied to the initial segmentation surface, and the segmentation on each section is further refined by a 2D level set. The parameters of the 3D and 2D level sets are presented in Table 1.

**Table 1.** Parameters for the Level Sets

Level Set	α	β	γ	*n*
3D	1	0.4	*q*	20
2D	4.0	0.2	0.5	10

**Figure 4.**
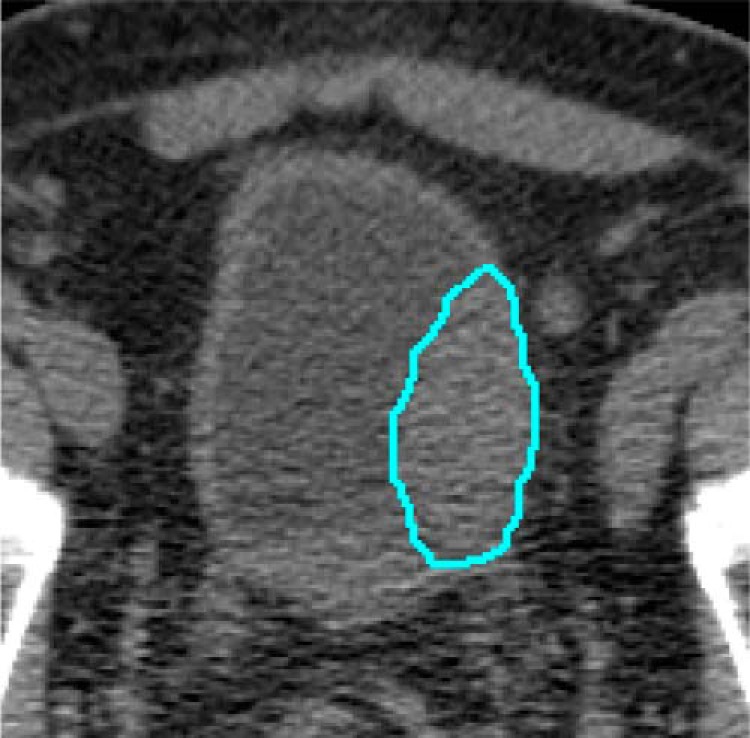
Bladder cancer segmentation on the CT section shown in Figure 1 using the bladder likelihood map shown in Figure 3.

The 3D level set brings the contour toward the sharp edges, and also expands it slightly in regions of low gradient. The parameter “*q*” in Table 1 is defined as a linear function σ*M* + λ of the 2D diagonal distance *M* of the VOI box in millimeters, where σ = 0.06, λ = −0.11 as shown previously (30). After the 3D level set refinement, a 2D level set is applied to every section of the 3D contours to further refine the contours. Details on the level sets used can be found in the literature (30). Figure 4 shows the final contour of the bladder cancer on the CT section from Figure 1 using the likelihood map shown in Figure 3.

### Evaluation Methods

Segmentation performance was evaluated by comparing the automatic segmentation results to the 3D hand-segmented contours. The average minimum distance and the Jaccard index (31) between the hand- and computer-segmented contours were calculated.

The average distance, *AVDIST*, is the average of the distances between the closest points of 2 contours and is calculated using the following equation:

(3) $$AVDIST\left(G,U\right)=\frac{1}{2}\left(\frac{\sum_{x\in G}\min\left\{d\left(x,y\right):y\in U\right\}}{N_{G}}+\frac{\sum_{y\in U}\min\left\{d\left(x,y\right):x\in G\right\}}{N_{U}}\right),\;\;$$

where *G* and *U* are the 2 contours being compared. N_G_ and N_U_ denote the number of voxels on *G* and *U*, respectively. The function *d* is the Euclidean distance. For a given voxel along the contour *G*, the minimum distance to a point along the contour *U* is determined. The minimum distances obtained for all points along *G* are averaged. This process is repeated by switching the roles of *G* and *U*. *AVDIST* is then calculated as the average of the 2 average minimum distances.

The Jaccard index is defined as the ratio of the intersection between the reference volume and the segmented volume to the union of the reference volume and the segmented volume, and calculated using the following equation:

(4) $$JACCARD^{3D}=\frac{V_{G}\cap V_{U}}{V_{G}\cup V_{U}},$$

A value of 1 indicates that *V*_*U*_ completely overlaps *V*_*G*_, whereas a value of 0 implies *V*_*U*_ and *V*_*G*_ are disjoint.

Receiver operating characteristics (ROC) analysis and area under the receiver operating characteristic curve (AUC) were used to estimate the accuracy for predicting T0 disease (complete response) after surgery based on the calculated change in GTV between pre- and post-treatment CT scans using the DL-CNN, the AI-CALS, and the manual segmentation methods. The AUCs from the radiologists' WHO criteria and RECIST estimates were also calculated.

## Results

**Figure 5.**
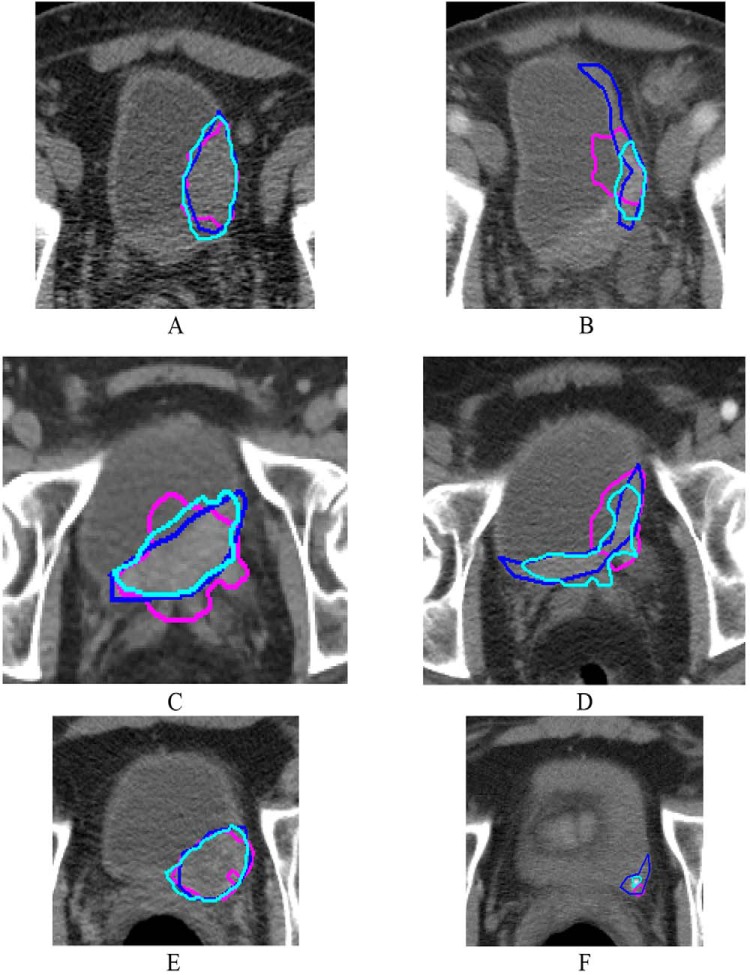
Examples of segmentations of bladder tumors in pre-treatment (A, C, E) and post-treatment (B, D, F) CT scans. The DL-CNN segmentation is shown in light blue. The auto-initialized cascaded level sets (AI-CALS) segmentation is shown in pink. The hand-outline is shown in dark blue. DL-CNN segmentation with AI-CALS segmentation and hand-outline for the cancer are shown in Figure 1 (A). Both computer methods segmented the lesion reasonably. The cancer lesion shrunk in size because of treatment, and became a part of the bladder wall (B). The DL-CNN undersegmented the cancer, not extending enough into the bladder wall. AI-CALS oversegmented the lesion, leaking into the bladder. The DL-CNN segmentation outlined the cancer relatively accurately, whereas the AI-CALS segmentation leaked (C). In this post-treatment scan, the cancer along the bladder wall was reasonably segmented by DL-CNN, whereas the AI-CALS was unable to follow the shape and leaked into the bladder (D). Both DL-CNN and AI-CALS segmented the bladder cancer reasonably well, but the AI-CALS slightly undersegmented the cancer (E). The bladder cancer responded to treatment, and thus, had shrunk considerably, making the segmentation difficult (F). Both the DL-CNN and the AI-CALS undersegmented the lesion.

Examples of DL-CNN-segmented bladder cancer on pre- and post-treatment CT scans, as well as the AI-CALS segmentation, are shown in Figure 5. The segmentation performance measures of both the DL-CNN and AI-CALS methods compared with reference standard 1 averaged over the pretreatment lesions, post-treatment lesions, and all lesions, as well as the *P* values from Student 2-tailed paired *t* test for the differences between the methods, are presented in Table 2. For all lesions, the difference in the average minimum distance was statistically significant with a *P* value of .001, whereas the difference in the Jaccard index approached significance with a *P* value of .058. The differences in the pretreatment lesion segmentation performances were statistically significant, with *P* < .001 and *P* < .015 for the average minimum distance and the average Jaccard index, respectively. The differences in the post-treatment lesion segmentation performances did not reach statistical significance.

**Table 2.** Segmentation Evaluation Using Reference Standard 1 (RS1)

	DL-CNN vs RS1	AI-CALS vs RS1	*P* Value
Average minimum distance *AVDIST*			
Pre-treatment	4.8 ± 2.3 mm	6.1 ± 3.6 mm	.001^a^
Post-treatment	4.6 ± 1.8 mm	4.9 ± 2.6 mm	.389
Both	4.7 ± 2.1 mm	5.5 ± 3.2 mm	.001^a^
Jaccard index *JACCARD*^3D^			
Pre-treatment	39.5 ± 17.1%	34.7 ± 15.8%	.015^a^
Post-treatment	32.6 ± 17.8%	32.7 ± 14.4%	.936
Both	36.3 ± 17.7%	33.8 ± 15.1%	.058

Abbreviations: DL-CNN, deep-learning convolution neural network; AI-CALS, autoinitialized cascaded level sets.

The results are shown in groups of pre-treatment, post-treatment, and both pre- and post-treatment lesions (126 lesions). The *P* values from Student 2-tailed paired *t* test for the differences between the DL-CNN and the AI-CALS segmentation methods are also shown. Some post-treatment lesions were determined to have shrunk completely by the radiologist; thus, no segmentation was performed.

^a^Statistically significant at *P* < .05.

The segmentation performance measures of the DL-CNN and AI-CALS methods compared with the 2 reference standards averaged over the pretreatment lesions, post-treatment lesions, and both pre- and post-treatment lesions for a subset of 29 cases are presented in Table 3. None of the differences reached statistical significance for this subset of cases.

**Table 3.** Segmentation Evaluation Between Hand-segmented Reference Standards (RS1, RS2) by 2 Different Readers for DL-CNN and AI-CALS Segmentation Methods for a Subset of 29 Cases

	DL-CNN vs RS1	AI-CALS vs RS1	DL-CNN vs RS2	AI-CALS vs RS2
Average minimum distance *AVDIST*				
Pre-treatment	4.8 ± 1.8 mm	5.3 ± 2.7 mm	4.9 ± 3.4 mm	4.5 ± 1.9 mm
Post-treatment	4.3 ± 1.7 mm	4.4 ± 1.8 mm	4.7 ± 3.1 mm	4.9 ± 3.7 mm
Both	4.6 ± 1.8 mm	4.8 ± 2.3 mm	4.8 ± 3.2 mm	4.7 ± 2.9 mm
Jaccard index *JACCARD*^3D^				
Pre-treatment	45.3 ± 8.5%	42.5 ± 14.1%	46.8 ± 9.3%	42.8 ± 12.5%
Post-treatment	29.8 ± 17.7%	32.9 ± 14.8%	28.8 ± 19.7%	28.6 ± 18.2%
Both	37.5 ± 15.8%	37.7 ± 15.2%	37.8 ± 17.8%	35.7 ± 17.1%

Abbreviations: DL-CNN, deep-learning convolution neural network; AI-CALS, autoinitialized cascaded level sets.

The segmentation evaluation results are for a subset of 29 cases divided into pre-treatment, post-treatment, and both pre- and post-treatment lesions (58 lesions). None of the paired differences between DL-CNN and AI-CALS reached statistical significance for this subset, probably because of the small sample size.

**Figure 6.**
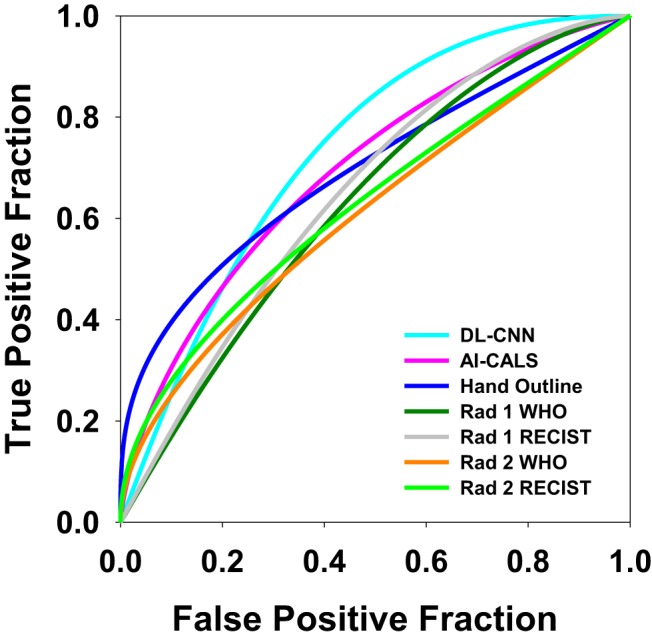
ROC curves for the prediction of complete response to chemotherapy. The area under the curves (AUCs) for GTV-based estimates were 0.73 ± 0.06 for DL-CNN, 0.70 ± 0.07 for AI-CALS, and 0.70 ± 0.06 for the radiologist's hand-outlines. The AUCs for the World Health Organization (WHO) criteria-based estimates were 0.63 ± 0.07 for radiologist 1 (Rad 1) and 0.61 ± 0.06 for radiologist 2 (Rad 2); the AUCs for the Response Evaluation Criteria In Solid Tumors (RECIST)-based estimates were 0.65 ± 0.07 for Rad 1 and 0.63 ± 0.06 for Rad 2.

Table 4 shows the AUC values for the different methods. The AUC for predicting complete response using GTV calculated using the DL-CNN segmentation achieved 0.73 ± 0.06, whereas that for the AI-CALS segmentation achieved 0.70 ± 0.07 compared with 0.70 ± 0.06 for the radiologist's hand-outline-based GTV. The differences between the 3 methods did not reach statistical significance. For the WHO criteria, the AUCs were 0.63 ± 0.07 and 0.61 ± 0.06 for the 2 radiologists. For the RECIST estimates, the AUCs were 0.65 ± 0.07 and 0.63 ± 0.06 for the 2 radiologists. Figure 6 compares the ROC curves for the different methods.

**Table 4.** AUC Values for Prediction of Cancer Stage pT0 After Surgery

Method	AUC
Volume	
DL-CNN	0.73 ± 0.06
AI-CALS	0.70 ± 0.07
Hand-outline	0.70 ± 0.06
WHO criteria	
Radiologist 1	0.63 ± 0.07
Radiologist 2	0.61 ± 0.06
RECIST	
Radiologist 1	0.65 ± 0.07
Radiologist 2	0.63 ± 0.06

Abbreviations: AUC, area under the receiver operating characteristic curve; DL-CNN, deep-learning convolution neural network; AI-CALS, autoinitialized cascaded level sets; WHO, World Health Organization; RECIST, Response Evaluation Criteria In Solid Tumors.

## Discussion

The results of this pilot study show that DL-CNN can be trained to segment bladder cancers in CT. The trained DL-CNN generates a likelihood map that identifies regions in the CT scans that are likely to be bladder cancers. By thresholding this map and performing minor refinement with level sets, we can obtain reasonable bladder cancer segmentations.

In our previous work on bladder segmentation with DL-CNN, level sets were also used to refine the output of the DL-CNN; as the contrast between the bladder and its surroundings is relatively high, cascaded level sets can refine the bladder boundary with high accuracy. On the other hand, with bladder cancer segmentation, the contrast between the tumor and the inside of the bladder can be much lower because the contrast material is often not used for the pre- and post-treatment CT. The level sets did not perform as well under these conditions, causing the segmentation to either leak or shrink incorrectly. Therefore, only a few iterations of the level sets were applied to the bladder cancer likelihood map to fill holes and smooth the segmentation.

The segmentation performance of the DL-CNN was better than that of AI-CALS in comparison with a radiologist's reference standard using the entire data set. The differences in the average minimum distance were statistically significant, whereas the difference in the Jaccard index approached significance (Table 2). When the data set is divided into pre- and post-treatment lesions, the DL-CNN performed significantly better than the AI-CALS for the pretreatment lesions, whereas the 2 methods performed comparably on the post-treatment lesions. The pre-treatment lesions are generally better defined than the post-treatment lesions. As the lesions change because of the treatment, the lesion generally shrinks and the boundaries become less distinct, making the post-treatment lesions more difficult to segment. Nevertheless, the changes in GTV estimated by the 2 computer methods were comparable to the estimates using radiologist's hand-outlines at prediction of complete response to treatment (Table 4).

The segmentation performances for the DL-CNN and the AI-CALS were compared with 2 reference standards in the subset that had both radiologists' manual outlines (Table 3). The results indicate that the performances of the 2 methods were consistent regardless of which reference standard was used.

The change in GTV estimated by the new DL-CNN segmentation method performed better than that by our previous AI-CALS system at prediction of complete response to treatment. Although the difference in the AUC for prediction of complete response to treatment did not reach statistical significance, probably because of the small data set, the segmentation performance results show that the segmentation by DL-CNN is better than that by AI-CALS.

Comparisons of the volume measurements with the WHO criteria and the RECIST show that the 3D measure of the bladder cancer (GTV) performs better than the 2D (WHO criteria) and the 1D (RECIST) measurements. The WHO criteria and the RECIST measurements performed worse compared with the GTV measures. This indicates that the 3D information (GTV) may be more reliable for assessment of bladder cancer treatment response.

There are limitations in this study. Although the data set was expanded compared with our previous study, the number of cases remained relatively small. Testing the method on a larger data set with wider ranges of sizes and types of bladder cancers will allow us to further validate the generalizability of the method. We will continue to enlarge the data set. Another limitation is that we used only 1 set of the radiologist's hand segmentations as the reference standard for the entire data set. To study the inter- and intraobserver variability in the hand segmentations of the bladder cancer, additional independent hand segmentations by different radiologists would be required.

Bladder cancer segmentation is important, as it defines the regions to be analyzed for the characterization of the lesion. We plan to expand our work to investigate if radiomic features extracted from the segmented bladder cancers, in conjunction with the GTV change, can improve the assessment of response to chemotherapy or other treatments. Although the DL-CNN method shows more promising results than the AI-CALS method at present, there remains more room for improvement on the segmentation performance of both methods, particularly for the post-treatment tumors. Further development and validation with a larger data set are also required to confirm the relative performance of the 2 approaches.

In conclusion, our results show that DL-CNN is useful for 3D segmentation of bladder cancers for a variety of bladder cancer shapes and sizes. The DL-CNN and the AI-CALS methods were able to automatically segment the cancers, with results similar to those of the radiologists' results. The 3D information from CT provides more accurate information on the changes in the tumor size in response to treatment compared with the 2D (WHO criteria) and 1D (RECIST) estimations used in current clinical practice. This study suggests that computerized segmentation of bladder cancers using DL-CNN has the potential to assist in the assessment of tumor volume and treatment response of bladder cancer by providing a more accurate 3D information without the extensive effort of manual segmentation.
